# Adjusting blood redistribution to suppress flow disturbances of hemodialysis arteriovenous fistula: a computational fluid dynamics analysis

**DOI:** 10.3389/fbioe.2025.1551993

**Published:** 2025-03-20

**Authors:** Yong-Jiang Li, Hui-Min Hou, Zheng Liu, Chun-Dong Xue, Jing-Tong Na, Qing-Mei Meng, Zhe-Yuan Li, Hai-Yang Sun, Yu-Lin Wu, Shu-Xin Liu, Kai-Rong Qin

**Affiliations:** ^1^ Institute of Cardio-Cerebrovascular Medicine, Central Hospital of Dalian University of Technology, Dalian, Liaoning, China; ^2^ School of Biomedical Engineering, Faculty of Medicine, Dalian University of Technology, Dalian, Liaoning, China; ^3^ School of Optoelectronic Engineering and Instrumentation Science, Dalian University of Technology, Dalian, Liaoning, China

**Keywords:** arteriovenous fistula, blood redistribution, computational fluid dynamics, flow disturbance, hemodialysis

## Abstract

**Introduction:**

The dramatic hemodynamic disturbances induced by arteriovenous fistula (AVF) creation are universally acknowledged as the triggering factors for AVF dysfunction. The postoperative blood redistribution is greatly relevant with the flow disturbances of the AVF, such as disturbed flow, low wall shear stress (WSS), and oscillating WSS. However, the relationship between blood redistribution and hemodynamic disturbances of AVF remains unexamined. The role of clinically observed retrograde blood flow at the distal radial artery is rarely understood.

**Methods:**

In this study, an idealized AVF model was developed with clinical data collected from end-stage renal disease patients. By considering the postoperative blood redistribution, the influence of the blood flow rate ratio on hemodynamic disturbances is numerically studied.

**Results and discussion:**

The results demonstrate that the creation of the AVF can result in flow disturbances such as vortex, reciprocating flow, and low and reciprocating WSS, whose occurrence regions are consistent with clinical observations. The flow rate ratio and flow direction of the distal radial artery play important roles in regulating the low-WSS area within the AVF anastomosis, especially for the flow rate of the proximal radial artery (PRA). Moreover, the clinically observed retrograde blood flow in the distal radial artery contributes to the reduction in the low-WSS area, revealing a compensatory mechanism. This study can provide valuable insights for understanding the effect of blood redistribution on flow disturbances in the AVF, as well as the compensatory role of the retrograde distal radial artery flow, which helps optimize blood redistribution for a well-functioning AVF.

## 1 Introduction

Hemodialysis is the most common treatment method for patients with end-stage renal disease (ESRD). To achieve efficient hemodialysis, a well-functioning vascular access (VA) is required (Bello et al., 2022; Shahinian et al., 2020). Typically, the radial–cephalic arteriovenous fistula (AVF) is the most preferred VA and strongly recommended by clinical guidelines worldwide due to its advantages of longevity, low complications, and low morbidity (Lok et al., 2020; Arhuidese et al., 2022). Despite being the optimal VA, significant primary failure rates and unsuccessful maturation rates have been reported (Shiu et al., 2019; Jia et al., 2015; Shiu et al., 2019; Huber et al., 2021; Venkat Ramanan et al., 2022).

It is universally acknowledged that the dramatic changes in hemodynamic effects after AVF creation are mainly relevant to AVF maturation failure. The resulting flow disturbances referring to the disturbed flow, low wall shear stress (WSS), and oscillating WSS are considered critical hemodynamic factors leading to neointimal hyperplasia (NIH) and further AVF maturation failure (Tordoir et al., 2018; Jia et al., 2015; Shiu et al., 2019; Colley et al., 2020; Hyde-Linaker et al., 2022; Ene-Iordache et al., 2015; Browne et al., 2015; Sadaghianloo et al., 2019). AVF bypasses the resistance vessels of the distal extremity and forms a short circuit between the arterial and the venous systems. As a result, the rate of volume flow through AVF increases dramatically (Colley et al., 2020; Dixon, 2006). The dramatic hemodynamic changes are greatly relevant to AVF anastomosis techniques, including anastomosis types, size, and angle and AVF configuration Ene-Iordache et al. (2015); Ene-Iordache and Remuzzi (2017); Rosado-Toro et al. (2022); Pike et al. (2017); Van Canneyt et al. (2010); Iori et al. (2015); Carroll et al. (2019); Alam et al. (2022); Suqin et al. (2020). In some cases, owing to the overall resistance balance after AVF creation, there will be retrograde flow of blood from the distal artery to feed the low-resistance vein (Ene-Iordache and Remuzzi, 2012; Colley et al., 2020; Dixon, 2006; Ramuzat et al., 2003; Hyde-Linaker et al., 2022; Sivanesan et al., 1998). Hence, understanding the role of postoperative blood redistribution in the flow disturbances of AVF is essential for probing the hemodynamic mechanisms of AVF maturation failure.

Computational fluid dynamics (CFD) is a common methodology that allows for reconstruction and visualization of the AVF hemodynamic microenvironment (Ene-Iordache and Remuzzi, 2012; Hyde-Linaker et al., 2022; Ene-Iordache et al., 2015; Ene-Iordache and Remuzzi, 2017; Rosado-Toro et al., 2022; Pike et al., 2017; Van Canneyt et al., 2010; Iori et al., 2015; Carroll et al., 2019; Alam et al., 2022; Suqin et al., 2020; Carroll et al., 2019; Yang et al., 2020). CFD with idealized geometry has the advantages of low computational cost and facilitation of parametric analysis. Various CFD studies were conducted with ideal AVF models to analyze different AVF surgical techniques and optimize flow pathways (Ene-Iordache and Remuzzi, 2012). An ideal end-to-side AVF model was proposed to study the influence of anastomosis size and angle on hemodynamic factors (Van Canneyt et al., 2010; Yang et al., 2020). The pressure decreased with a larger anastomosis cross-sectional area and an angle wider than 43°. An anastomosis angle exceeding 58° would lead to a retrograde flow of blood from the distal artery. The effects of the arterial curvature on blood flow and oxygen transport patterns within the AVF were also investigated (Iori et al., 2015). The results revealed that a placing a vein graft onto the outer curvature of a curved artery would avoid unsteady flow and placing a vein graft onto a straight artery or the inner curvature of a curved artery would prevent low WSS and low lumen-to-wall oxygen flux that leads to the development of NIH. Some numerical studies (Ene-Iordache and Remuzzi, 2012; 2017; Remuzzi and Ene-Iordache, 2013) have been conducted to characterize the patterns of disturbed flow and WSS associated with stenosis and vascular remodeling, including the low and oscillating WSS as well as the multidirectional and reciprocating near-wall flow. The CFD simulations with an end-to-side configuration showed good agreement with experimental studies (Colley et al., 2020; Remuzzi and Ene-Iordache, 2013; Gunasekera et al., 2020). These abovementioned CFD studies elucidate complex hemodynamic factors related to AVF outcomes (NIH, stenosis, and thrombosis), which helps understand the etiology of AVF maturation failure. However, how post-operative blood redistribution affects flow disturbances in AVF remains unexamined. The role of clinically observed retrograde blood flow at the distal radial artery is not well understood.

In the present study, a CFD study is conducted to analyze the effect of blood redistribution on the flow disturbance of the AVF. An ideal AVF model is developed using realistic clinical data collected from ESRD patients. By considering the blood redistribution induced by AVF creation in terms of varying flow rate ratios, the hydrodynamic disturbances in the AVF are numerically studied, including flow patterns and abnormal areas with low and reciprocating WSS. Moreover, the role of retrograde blood flow at the distal radial artery is identified.

## 2 Materials and methods

### 2.1 Acquisition of clinical data

**FIGURE 1:**
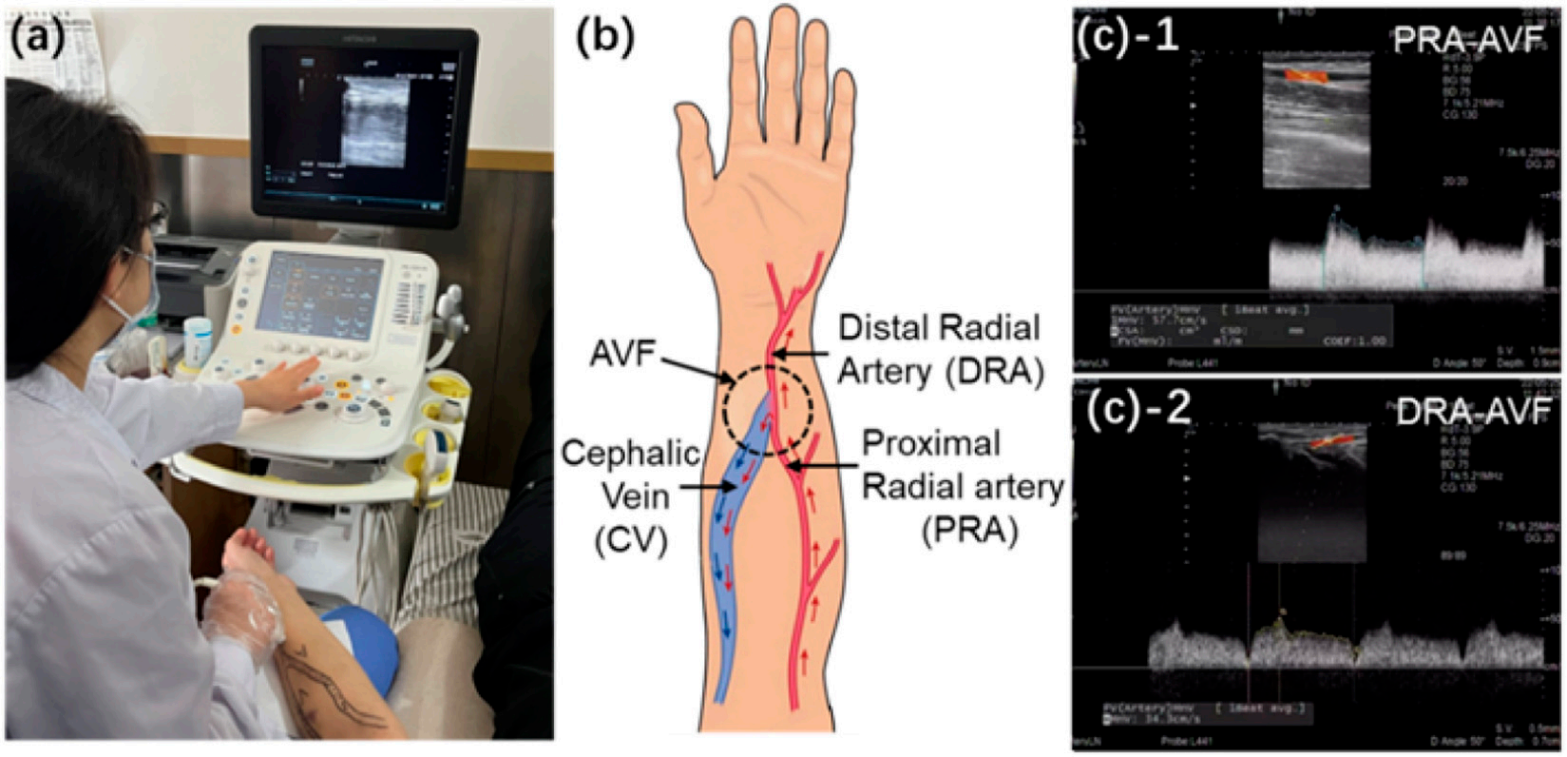
**(a)** Real image during ultrasonic acquisition of blood flow velocity. **(b)** Schematic of radial–cephalic AVF. **(c)** Clinical measurements of blood flow velocities at the PRA and DRA of AVF and RA and CV of the normal forearm.

A female patient undergoing maintenance hemodialysis for 13 weeks was included in the trial and data collected. An end-to-side anastomosis was surgically created to construct a radiocephalic AVF for hemodialysis access (Figure 1). The waveforms of blood flow velocities at the proximal radial artery (PRA) and distal radial artery (DRA) of the AVF in the left forearm were measured using the Doppler ultrasonic detector (ARIETTA 60, Hitachi, Japan). The direction of blood flow (antegrade or retrograde) in the DRA is determined by the color (red or blue) of the ultrasound image for identification. Simultaneously, the diameters of the PRA, DRA, and distal cephalic vein (DCV) were measured 4 cm away from the anastomosis (Supplementary Table S1). The blood pressure at the distal cephalic vein is evaluated using the invasive pressure sensor (TRAM 451, GE Healthcare, United States). The absolute pressure at the cephalic vein is ∼ 8 mmHg (1,064 Pa). The heart rate of the patient is measured using a blood pressure monitor with an average value of ∼ 70 bpm. The procedure was approved by the Ethics Committee of the Affiliated Central Hospital of Dalian University of Technology.

### 2.2 Geometric model and CFD simulation

**FIGURE 2:**
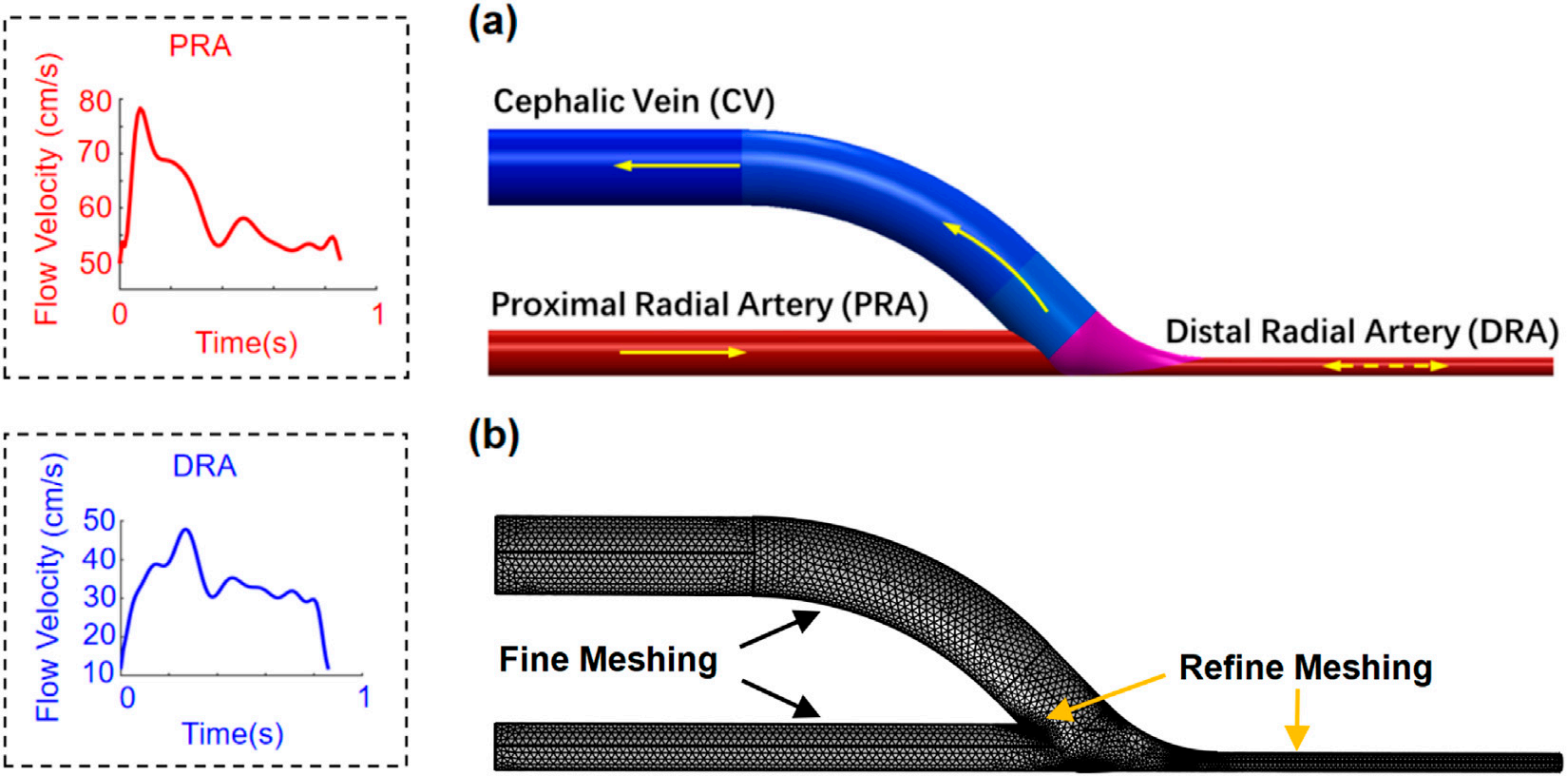
Geometry **(a)** and meshing **(b)** of the patient-specific idealized AVF model. Flow velocities at the PRA and DRA are presented in one cardiac cycle.

An idealized end-to-side AVF geometry (Figure 2) is reconstructed based on the measured data of the patient (Table 1). Two lumens with diameters of 3.0 mm and 1.2 mm are idealized accordingly as PRA and DRA by aligning their outer edges. A lumen with a diameter of 5.0 mm is modeled as the cephalic vein. The anastomotic segment is merged smoothly using a basis spline curve with an anastomosis angle of 45 ° , and the distal vein segment is positioned parallel with the artery segment in the longitudinal axis of the forearm.

**TABLE 1:** Average velocity and diameter of the PRA, DRA, and CV at the AVF forearm.

Vessel	Average velocity (cm/s)	Diameter (mm)
PRA	57.7	3.0
DRA	34.3	1.2
CV	——	5.0

The idealized AVF model was reconstructed using SOLIDWORKS 2021 and further imported into the simulation software COMSOL Multiphysics^®^ for analysis. To conduct the simulation, the boundary conditions were defined according to the clinical acquisition data. A uniform velocity condition (Figure 2) extracted from the ultrasonic image (Figure 1) was applied at the PRA inlet of the AVF model. At the DRA, a uniform velocity condition (Figure 2), referring to the same velocity across the entire cross-section, extracted from the ultrasonic image (Figure 1), was set, where the flow direction depends on the anterograde or retrograde flow of blood. A uniform pressure condition, referring to the same pressure across the entire cross-section, was adopted at the CV outlet. Infinite conditions were added to extend the PRA, DRA, and CV segments to allow sufficient length for flow to fully develop prior to entering the AVF anastomosis. Slip-free boundary conditions (the no-slip hypothesis that the fluid velocity at the wall is 0) were adopted at the walls of blood vessels. A mesh comprising ∼ 0.12 million elements was required to achieve a grid-independent solution (details in Supplementary Figure S1). Simulations were conducted within three cardiac cycles with a time step of 0.01 s by balancing the computational cost and accuracy (Ene-Iordache and Remuzzi, 2012; Carroll et al., 2019; Jodko et al., 2017). Owing to the low Reynolds number of ∼ 445, the laminar flow module is applied for CFD analysis. The steady-state solutions at specific intervals in the third cardiac cycle (1.72–2.58 s) were extracted for data analysis, including the moments of maximum ( ∼ 1.82 s), average ( ∼ 2.02s), and minimum ( ∼ 2.58 s) flow velocities.

The creation of the AVF results in blood redistribution along the blood circulation circuits of the upper limb. The blood flow from the left ventricle to the PRA bifurcates into two main circuits: the first circuit goes to the upper extremity, then drains into the vein of the upper limb, and finally back to the right atrium, and the other circuit passes through the AVF anastomosis, then flows into the CV, and returns to the right atrium. As a result, the total flow rate of the PRA (defined as $Q_{PA}$ ) and DRA (defined as $Q_{DA}$ ) varies depending on the resistance and compliance of the upper limb vessels, the operative anastomosis area, the diameters of vessels, etc. Herein, we characterize the effect of blood redistribution that is caused by AVF creation on the flow disturbance of AVF. By varying the flow rate ratio $\gamma_{i}$ (i = 1,2,3,4) (Table 2), the variation in the low WSS area is studied. Considering the anterograde and retrograde flow of blood through the DRA observed in clinic, the flow direction of the DRA is changed accordingly (Table 2). It should be noted that the clinically acquired flow velocities of the PRA and DRA are used as the references, with ϵ=100% for $\gamma_{i}$ (i = 1,2,3).

**TABLE 2:** Simulation conditions of AVF blood redistribution characterized by the flow rate ratio $\gamma_{i}$ (i = 1,2,3,4).

Flow rate ratio $\gamma_{i}$	Flow rate of PRA	Flow rate of DRA	Flow direction of DRA	Coefficient ε
$\gamma_{1}$ = $Q_{DA}\;/\;\varepsilon Q_{PA}$	$\varepsilon Q_{PA}$	$Q_{DA}$	Anterograde	70%, 80%, 90%,100%, 110%, 120%, and 130%
$\gamma_{2}$ = $\varepsilon Q_{DA}\;/\;Q_{PA}$	$Q_{PA}$	$\varepsilon Q_{DA}$		
$\gamma_{3}$ = $\varepsilon Q_{DA}\;/\;\varepsilon Q_{PA}$	$\varepsilon Q_{PA}$	$\varepsilon Q_{DA}$		
$\gamma_{4}$ = $\varepsilon Q_{DA}\;/\;Q_{PA}$	$Q_{PA}$	$\varepsilon Q_{DA}$	Retrograde	10%, 20%, 30%, 40%, and 50%

### 2.3 Post-processing of simulation results

**FIGURE 3:**
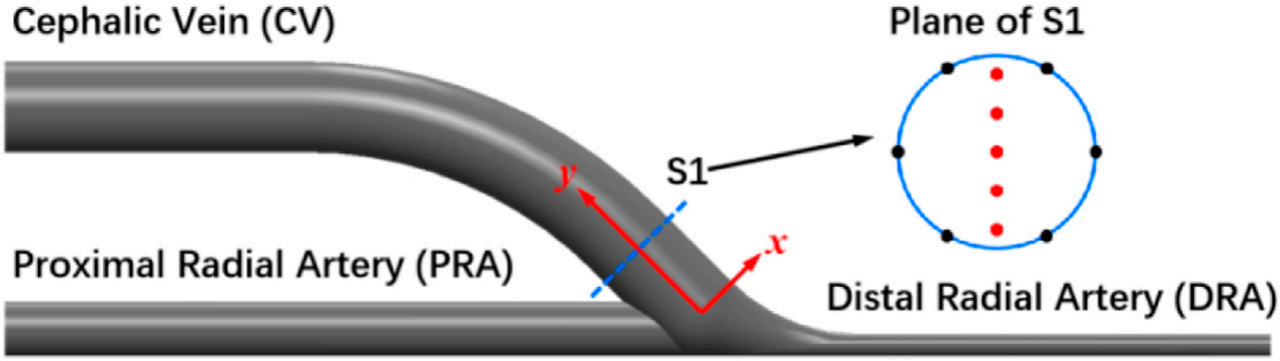
Observation cross-sections at post-anastomosis (S1).

After CFD simulations, the distributions of flow velocity, streamline, and WSS were exported. A coordinate system (Figure 3) is defined with the origin at the center of anastomosis. The y-axis is parallel to the direction of the proximal vein. The x-axis is in the positive direction to the outer edge of the anastomosis. To characterize the flow disturbance, the streamlines are displayed at the cross-section S1 near the anastomosis, with their normal vectors parallel to the y-axis. The evolutions of flow velocity at five evenly spaced points (red dots) are observed at the vortex region (Figure 3). To characterize the WSS, the distribution of the WSS magnitude is calculated across the whole computational domain, and the distribution of WSS is observed at the cross-section S1 around the vortex region. The evolutions of WSS at six evenly spaced points (black dots) are observed (Figure 3). At different flow rate ratios $\gamma_{i}$ (i = 1,2,3,4), the areas of low WSS regions are normalized by the results calculated with the conditions observed in the clinic, as indicated in Supplementary Table S1.

## 3 Results

### 3.1 Distribution and evolution of flow patterns at the AVF anastomosis

**FIGURE 4:**
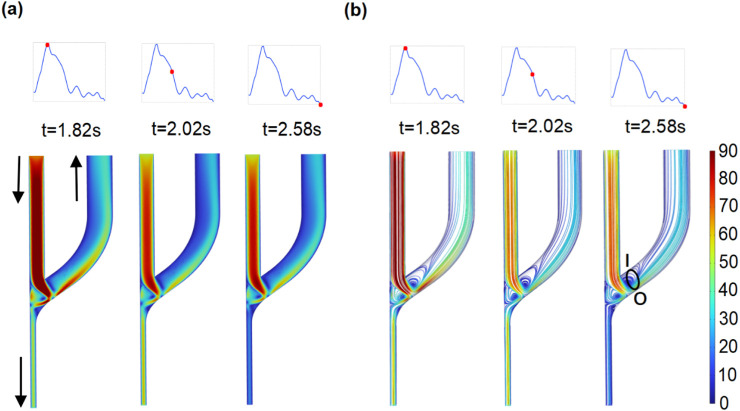
Distribution of flow velocity and streamline at the whole domain of the AVF model. **(a)** Flow velocity (cm/s). **(b)** Distribution of streamlines.

The flow patterns and disturbances in the AVF are numerically characterized. The distributions of blood flow velocity are shown according to the intervals of maximum, average, and minimum velocities (Figure 4). In all cases, the regions of high flow velocity emerge, in which flow from the PRA normally to the outer edge of the anastomosis is observed (Figure 4A). The high-velocity stream separates two areas with low flow velocity and vortex flow. As indicated in Figure 4B, the distribution of streamlines clearly reflects two vortex areas located on both sides of the high-velocity stream. One emerges above the high-velocity stream and appears at the inner edge of the anastomosis to the CV end, while another emerges below the high-velocity stream and appears at the anastomosis opening to the PRA end. The findings are consistent with clinical observations and previous results (Colley et al., 2020; Iori et al., 2015; Remuzzi and Ene-Iordache, 2013).

**FIGURE 5:**
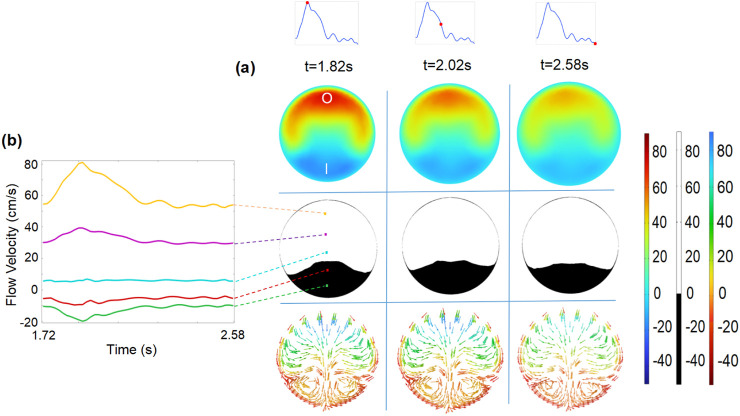
**(a)** Flow velocity ( $1^{st}$ row) and binary image of flow velocity ( $2^{nd}$ row) at the plane of S1, streamline ( $3^{rd}$ row) for time intervals of 1.82 s, 2.02 s, and 2.58 s, respectively. **(b)** Evolution of flow velocity at five evenly spaced points along the S1 centerline.

Within the vortex region, the topology of streamlines at the plane of S1 (Figure 5) is displayed. At selected time intervals, the area of the negative velocity opposite to the y-axis appears at the inner edge of plane S1 and varies with the flow velocity (Figure 5). Simultaneously, the circular current of streamlines reveals the existence of the vortex, which varies in size depending on the pulsatile blood flow velocity. At this specific section of the vortex, the evolutions of flow velocities are observed at five evenly spaced points of plane S1 (left subfigure in Figure 5). The alternatively positive and negative variations from the outer to inner edge of plane S1 probe once again the existence of the vortex, which is associated with the development of NIH (Remuzzi and Ene-Iordache, 2013; Cunnane et al., 2019).

### 3.2 Distribution and evolution of WSS at the AVF anastomosis

**FIGURE 6:**
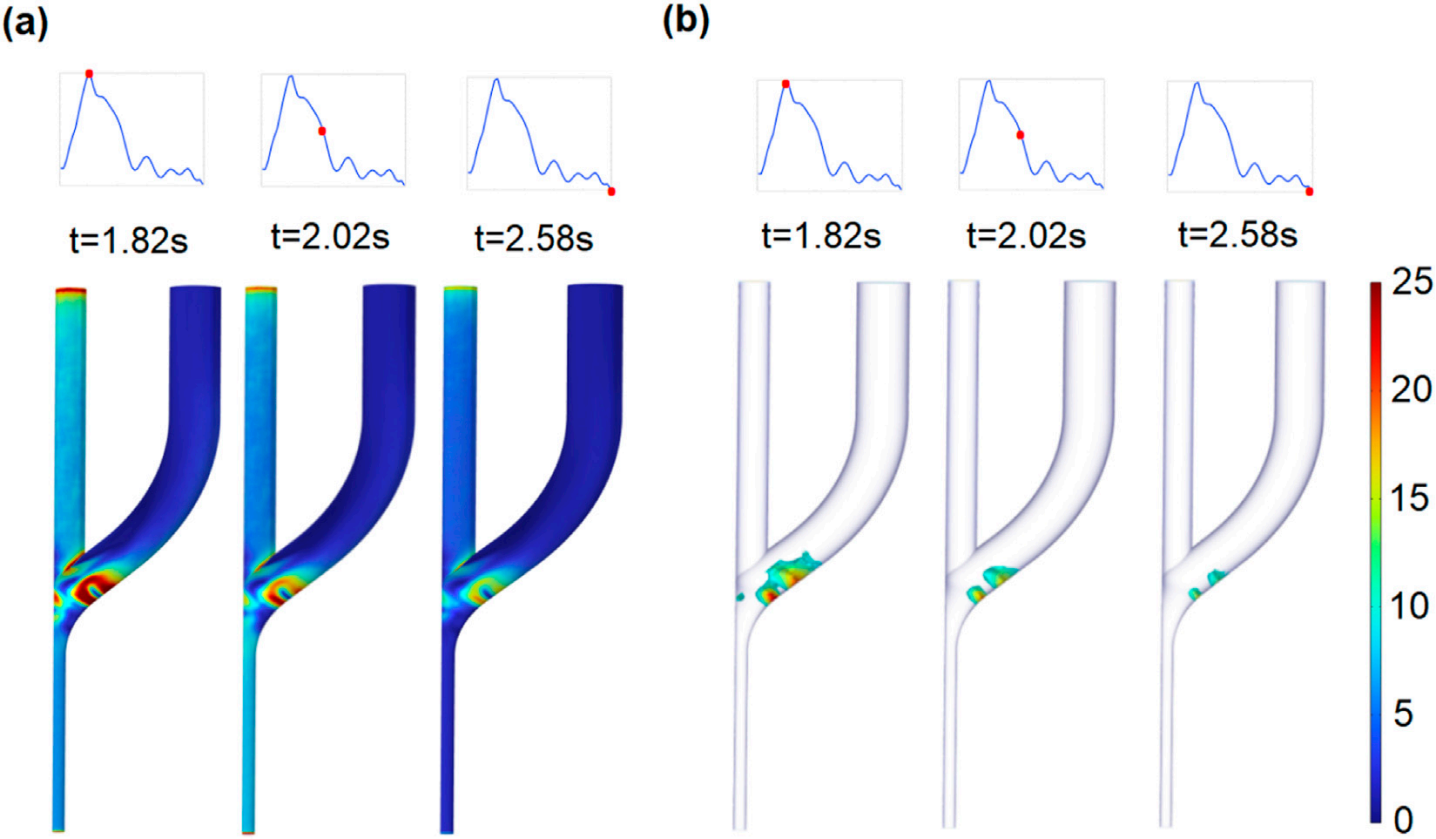
Distribution of wall shear stress **(a)** and high wall shear stress ( ≥ 10 Pa) within AVF **(b)**.

The WSS is the critical hemodynamic factor associated with maintaining the function of the developed AVF. Therefore, the WSS values at the vessel walls of the AVF (Figure 6A) are analyzed. The high WSS ( ≥ 10 Pa) is distributed mostly at the PRA and the anastomosis (Figure 6B) due to the high flow rate. The maximum WSS ( ≥ 20 Pa) merges at the anastomosis opening to the outer edge of the proximal CV where the high-velocity stream impacts, as shown in Figure 4.

**FIGURE 7:**
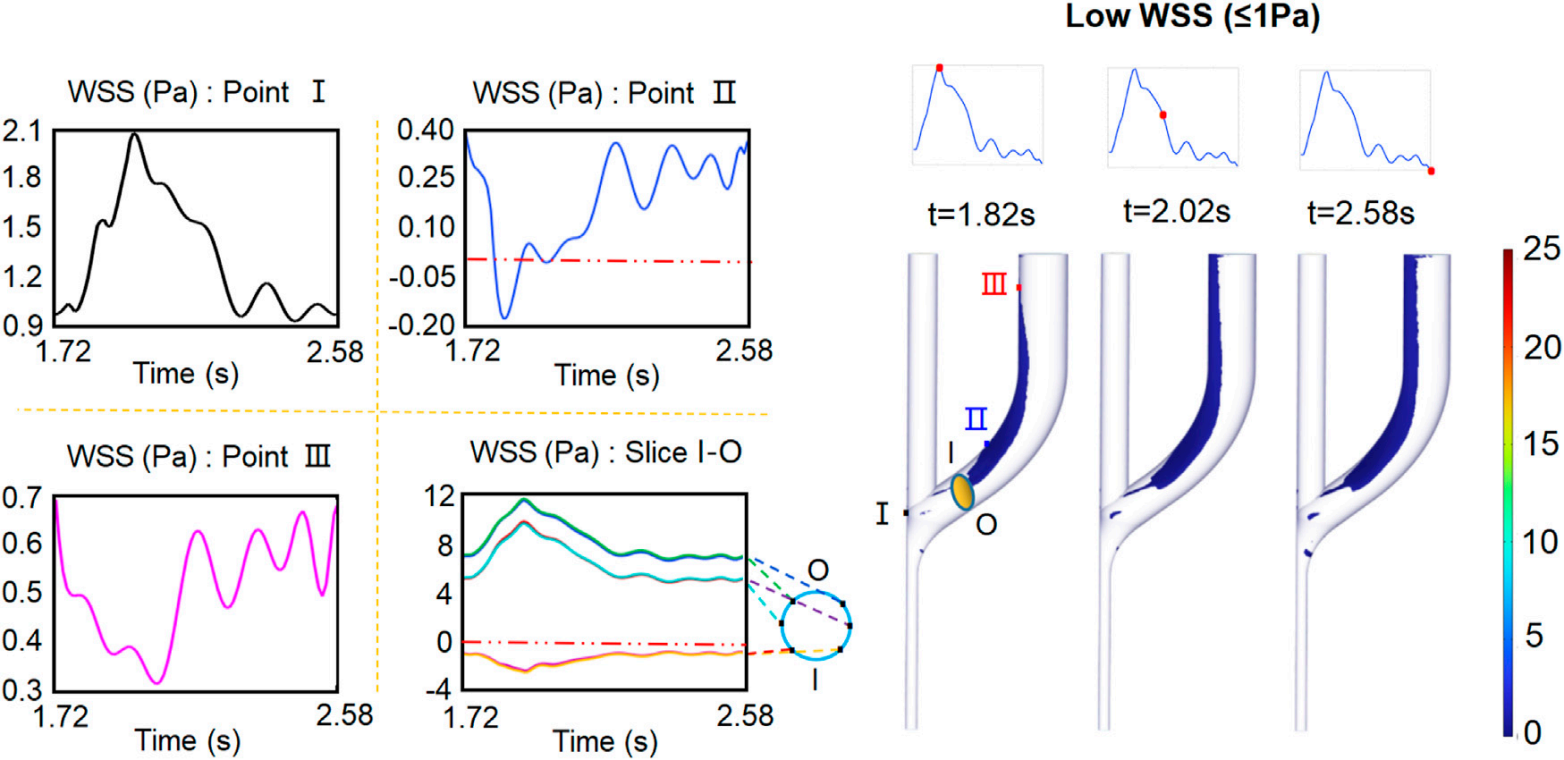
Distribution of low WSS ( ≤ 1 Pa) within the AVF.

The relatively low WSS ( ≤ 1 Pa) is located primarily at the inner edge of the proximal vortex area extending to the distal CV. As the flow velocity decreases, the low WSS area significantly enlarges and propagates to the distal CV. The evolutions of WSS at the wall of plane S1 are monitored at six observation points. Similar to Point I, in all points in plane S1, although the directions of the WSS waveforms are not exactly the same, the variation tendencies are similar, which exhibit higher WSS magnitudes at the outer wall compared with those at the inner wall (Figure 7). Interestingly, close to the inner wall of the low-WSS region, the WSS shows an oscillating (such as Point III) and oscillating and reciprocating patterns (such as Point II).

### 3.3 Effect of blood redistribution on low-WSS areas

**FIGURE 8:**
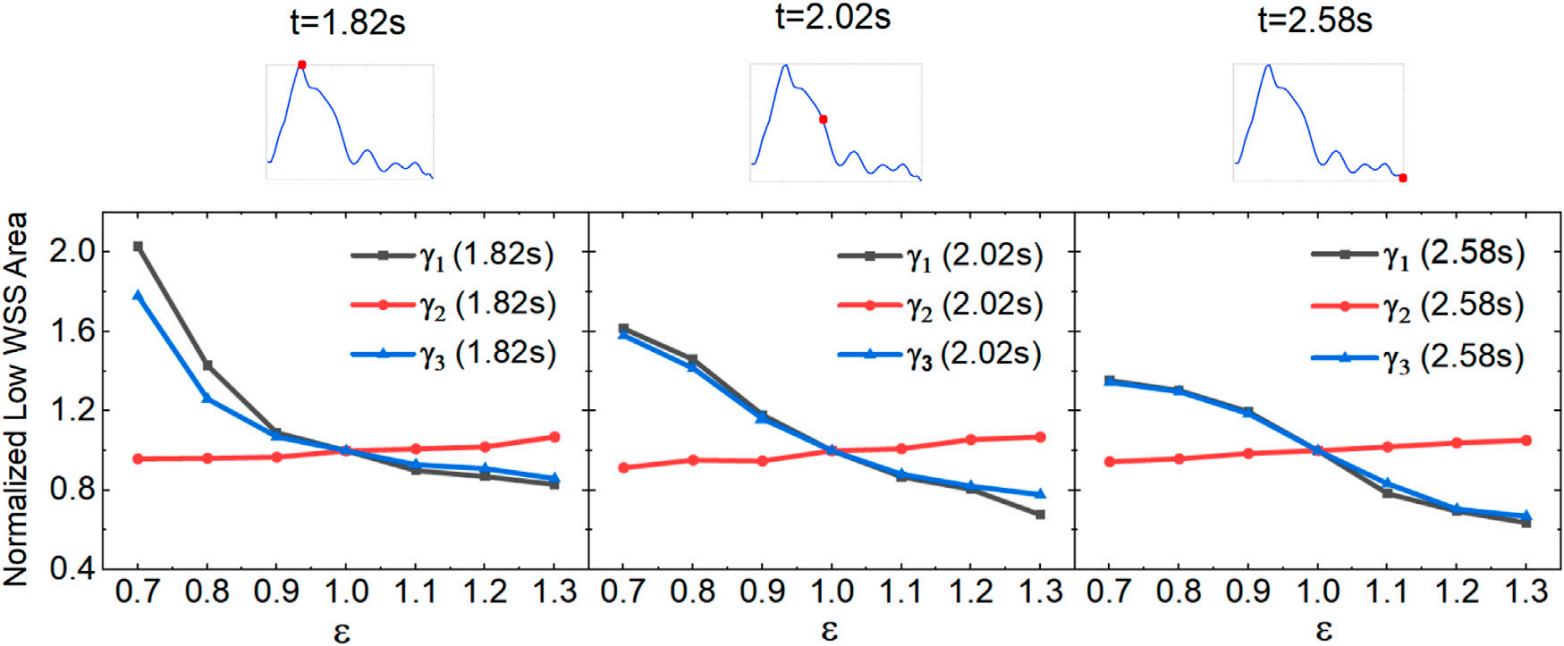
Variation of low-WSS areas at different flow rate ratios for the anterograde DRA blood flow.

Blood redistribution resulting from AVF creation varies with each individual and surgical method, ultimately leading to the change in the blood flow rate and the DRA ratio. Herein, we characterize the effect of the flow rate ratio $\gamma_{i}$ (i = 1,2,3,4) on the area of low WSS. As shown in Figure 8, when $Q_{DA}$ is kept constant, the low-WSS area decreases significantly with the increase in $\varepsilon \;Q_{PA}$ . In contrast, when $Q_{PA}$ is kept constant, the low-WSS area varies slightly with $\varepsilon Q_{DA}$ ; a 30 % increase in the flow rate of the DRA only leads to 10 % increase in the low-WSS area. When both $Q_{PA}$ and $Q_{DA}$ change simultaneously by ε , the low-WSS area varies significantly and inversely with ε , showing a comparable tendency as the case of changing the flow rate of PRA alone. These findings reveal that the flow rate of the PRA plays an important role in affecting the low-WSS area compared to the flow rate of the DRA. At different intervals in a circle, the tendencies of the low WSS area at the flow rate ratio $\gamma_{i}$ (i = 1,2,3) are consistent. The low-WSS area is directly proportional to the value of the flow rate.

### 3.4 Effect of retrograde DRA flow on flow patterns and WSS

**FIGURE 9:**
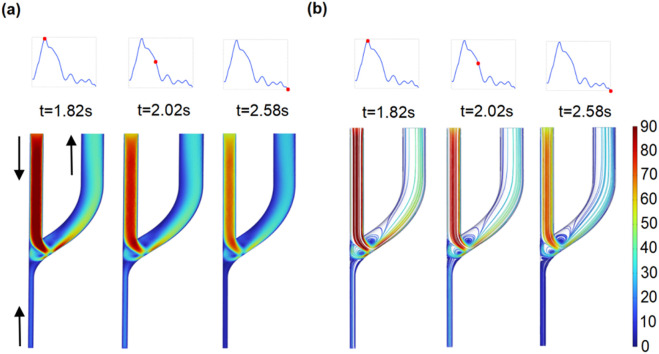
Distribution of flow velocity **(a)** and streamline **(b)** within AVF for the retrograde DRA blood flow ( $\gamma_{4}$ = 0.3).

To characterize the role of clinically observed retrograde DRA flow, the flow rate of the PRA is kept constant; the distributions of the blood flow velocity and the corresponding streamlines are shown in Figure 9. The blood flow distributions and the vortex locations are similar to those observed in the anterograde blood flow case. Interestingly, the area of the two vortex regions at the peri-anastomosis slightly decreases due to the increase in the total flow rates in the venous segment.

**FIGURE 10:**
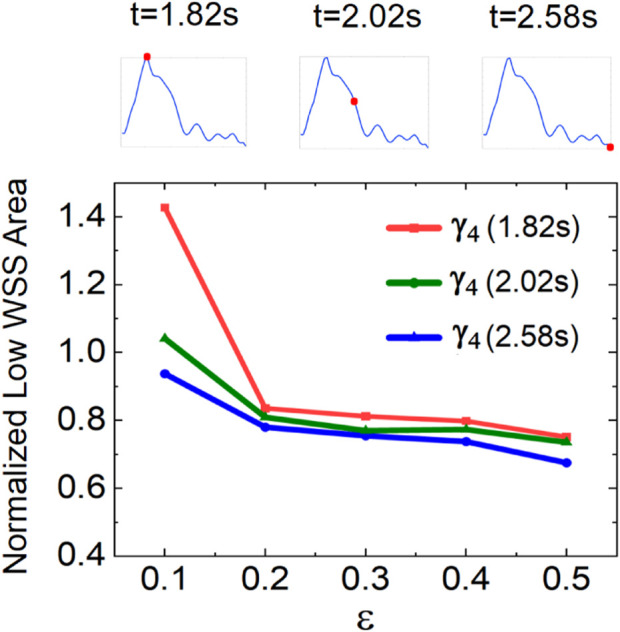
Variation in low-WSS areas at different flow rate ratios in the retrograde DRA blood flow.

To quantify the varying retrograde DRA flow on the low-WSS area, we measured the low-WSS area at different flow rate ratios $\gamma_{4}$ (Figure 10). Within a wide range of $\varepsilon Q_{DA}$ (0.2 ≤ε≤ 0.5), the area of low WSS remains at 0.8 and slightly decreases with ε . The results indicate that the retrograde DRA flow can contribute to the reduction in the area of the low-WSS region in the AVF anastomosis. The low-WSS area is 80 % of that in the reference case. When the flow rate decreases to 0.1 $Q_{DA}$ , a rapid increase in the area of low WSS is observed. This is because the low-WSS region within the DRA owing to its low flow rate.

## 4 Discussion

The radiocephalic AVF is the optimal VA for hemodialysis. However, it suffers from high rates of early-maturation failure and peri-anastomotic restenosis owing to the dramatic hemodynamic disturbances after AVF creation (Colley et al., 2020; Dixon, 2006). In this study, an idealized end-to-side AVF model was constructed to analyze the effects of blood redistribution on AVF hemodynamic disturbances. The simulation results demonstrated the existence of the vortex, reciprocating flow, and low and reciprocating WSS around the AVF anastomosis (Figures 4–7); their occurrence regions are in agreement with clinical observations and previous results (Jia et al., 2015; Ene-Iordache and Remuzzi, 2012; Hyde-Linaker et al., 2022; Van Canneyt et al., 2010). The complex hemodynamic disturbances in the anastomotic region are proven to be highly prone to the AVF complications such as NIH and vascular stenosis (Colley et al., 2020). Therefore, understanding the hemodynamic disturbance after AVF creation and its related factors is essential for avoiding further AVF complications.

In the simulations, we have quantitatively characterized the low-WSS region, which is a critical hemodynamic factor for maintaining the AVF function. The dependence of the low-WSS area on the flow rate ratio of the PRA to DRA and the DRA flow direction was analyzed. The significant roles of the PRA flow rate and retrograde DRA flow in reducing the area of the low-WSS regions were revealed (Figures 8–10). For this reason, the optimization of blood redistribution within AVF can be beneficial to reduce the low-WSS area, especially for high PRA flow rate and retrograde DRA flow. Therefore, in surgical operations (Farber et al., 2016) or hemodialysis, the resistance and compliance of both extremities and distal CV should be considered (Sadaghianloo et al., 2019; Remuzzi and Ene-Iordache, 2013) to regulate the postoperative blood redistribution by enlarging the anastomotic opening and optimizing the artery-to-vein configuration (Gunasekera et al., 2021; Farrington et al., 2020; Shahverdyan et al., 2021). From another perspective, sports exercise and external counterpulsation are well-recognized non-invasive mechanical methods for improving the blood redistribution (Chen et al., 2023). Accordingly, appropriate exercise or counterpulsation intervention can regulate the blood redistribution after AVF creation, which may be an alternative non-invasive approach to reduce hemodynamic disturbance for a long-term AVF function (Wang et al., 2018; Andrade et al., 2021).

In clinical practice, the retrograde blood flow of the DRA is frequently observed and has been reported in previous studies (Ene-Iordache and Remuzzi, 2012; Colley et al., 2020; Dixon, 2006; Ramuzat et al., 2003; Hyde-Linaker et al., 2022; Sivanesan et al., 1998). However, the role of the retrograde DRA blood flow is rarely considered. Some studies (Ene-Iordache and Remuzzi, 2012; Colley et al., 2020; Sivanesan et al., 1998) demonstrated that the retrograde blood flow of the DRA is a result of the overall resistance balance in the newly created vascular access. Our findings suggest that the retrograde DRA flow can contribute to the reduction in the low-WSS region in the AVF anastomosis, which is the well-known risk factor for NIH formation and stenotic lesions. The retrograde DRA flow is beneficial to the vascular microenvironment. This finding reveals that the occurrence of retrograde blood flow at the DRA may be a compensatory mechanism for optimizing blood redistribution and improving the hydrodynamic microenvironment. Several studies have demonstrated that optimizing the AVF hemodynamic microenvironment can promote AVF maturation by regulating surgical techniques, anastomosis size and angle, and AVF graft design (Van Canneyt et al., 2010; Iori et al., 2015). Owing to individual differences in blood circulation among patients, it is still challenging to adjust the blood redistribution in the real AVF. Based on our findings, enhancing retrograde blood flow of the DRA would contribute to AVF hemodynamic micro-environment AVF maturation. Therefore, upper limb and handgrip exercises can be a good strategy to enhance the retrograde flow of the DRA for post-operative AVF maturation (Chen et al., 2023; Nantakool et al., 2022).

It should be noted that the proposed idealized AVF model is developed under the rigid wall assumption. The rigid model would fail to recognize the effect of circumferential stress induced by pulsating blood pressure (Rangel et al., 2023). As vascular remodeling during AVF maturation is affected simultaneously by WSS and blood pressure, the elasticity of blood vessels should be further considered in future research.

## 5 Conclusion

In this study, an idealized end-to-side AVF model is reconstructed using clinically measured data. The effect of blood redistribution on the flow disturbance of the AVF has been investigated by CFD analysis. The results indicated that flow vortex, reciprocating flow, and low and reciprocating WSS result from the creation of the AVF, and their occurrence regions are consistent with clinical observations. The hydrodynamic disturbances significantly depend on the flow rate ratio of the PRA to DRA and the DRA flow direction. The flow rate of the PRA plays an important role in affecting the low-WSS area. The retrograde DRA flow can contribute to the reduction of low-WSS region in the AVF anastomosis, revealing a compensatory mechanism. The findings provide essential information for understanding the hydrodynamic changes after AVF creation and the compensatory role of retrograde distal radial artery flow, which helps optimize blood redistribution to reduce the flow disturbance of the AVF.

## Data Availability

The original contributions presented in the study are included in the article/Supplementary Material; further inquiries can be directed to the corresponding authors.

## References

[B1] Alam N. Walsh M. Newport D. (2022). Experimental evaluation of a patient specific brachio-cephalic arterio venous fistula (avf): velocity flow conditions under steady and pulsatile waveforms. Med. Eng. and Phys. 106, 103834. 10.1016/j.medengphy.2022.103834 35926957

[B2] Andrade F. P. Nolasco T. Knorst M. M. Eidt Rovedder P. M. (2021). Aerobic exercise increases vascular diameter of arteriovenous fistula in hemodialysis patients. Blood Purif. 51, 732–738. 10.1159/000519880 34801998

[B3] Arhuidese I. J. Cooper M. A. Rizwan M. Nejim B. Malas M. B. (2022). Vascular access for hemodialysis in the elderly. J. Vasc. Surg. 69, 517–525.e1. 10.1016/j.jvs.2018.05.219 30683199

[B4] Bello A. K. Okpechi I. G. Osman M. A. Cho Y. Htay H. Jha V. (2022). Epidemiology of haemodialysis outcomes. Nat. Rev. Nephrol. 18, 378–395. 10.1038/s41581-022-00542-7 35194215 PMC8862002

[B5] Browne L. D. Bashar K. Griffin P. Kavanagh E. G. Walsh S. R. Walsh M. T. (2015). The role of shear stress in arteriovenous fistula maturation and failure: a systematic review. Nat. Rev. Nephrol. 10, e0145795. 10.1371/journal.pone.0145795 PMC4696682 26716840

[B6] Carroll J. Varcoe R. L. Barber T. Simmons A. (2019). Reduction in anastomotic flow disturbance within a modified end-to-side arteriovenous fistula configuration: results of a computational flow dynamic model. Nephrology 24, 245–251. 10.1111/nep.13219 29314372

[B7] Chen J.-W. Fu H.-Y. Hii I.-H. Tseng H.-W. Chang P.-Y. Chang C.-H. (2023). A randomized trial of postoperative handgrip exercises for fistula maturation in patients with newly created wrist radiocephalic arteriovenous fistulas. Kidney Int. Rep. 8, 566–574. 10.1016/j.ekir.2022.12.019 36938082 PMC10014374

[B8] Colley E. Simmons A. Varcoe R. Thomas S. Barber T. (2020). Arteriovenous fistula maturation and the influence of fluid dynamics. Proc. Inst. Mech. Eng. H. 234, 1197–1208. 10.1177/0954411920926077 32597295

[B9] Cunnane C. V. Cunnane E. M. Moran D. T. Walsh M. T. (2019). The presence of helical flow can suppress areas of disturbed shear in parameterised models of an arteriovenous fistula. Int. J. Numer. Method. Biomed. Eng. 35, e3259. 10.1002/cnm.3259 31483945

[B10] Dixon B. S. (2006). Why don’t fistulas mature? Kidney Int. 70, 1413–1422. 10.1038/sj.ki.5001747 16883317

[B11] Ene-Iordache B. Remuzzi A. (2012). Disturbed flow in radial-cephalic arteriovenous fistulae for haemodialysis: low and oscillating shear stress locates the sites of stenosis. Nephrol. Dial. Transpl. 27, 358–368. 10.1093/ndt/gfr342 21771751

[B12] Ene-Iordache B. Remuzzi A. (2017). Blood flow in idealized vascular access for hemodialysis: a review of computational studies. Cardiovasc. Eng. Technol. 8, 295–312. 10.1007/s13239-017-0318-x 28664239

[B13] Ene-Iordache B. Semperboni C. Dubini G. Remuzzi A. (2015). Disturbed flow in a patient-specific arteriovenous fistula for hemodialysis: multidirectional and reciprocating near-wall flow patterns. J. Biomech. 48, 2195–2200. 10.1016/j.jbiomech.2015.04.013 25920898

[B14] Farber A. Imrey P. B. Huber T. S. Kaufman J. M. Kraiss L. W. Larive B. (2016). Multiple preoperative and intraoperative factors predict early fistula thrombosis in the hemodialysis fistula maturation study. J. Vasc. Surg. 63, 163–170.e6. 10.1016/j.jvs.2015.07.086 26718822 PMC4698902

[B15] Farrington C. A. Robbin M. L. Lee T. Barker-Finkel J. Allon M. (2020). Early predictors of arteriovenous fistula maturation: a novel perspective on an enduring problem. J. Am. Soc. Nephrol. 31, 1617–1627. 10.1681/asn.2019080848 32424000 PMC7351000

[B16] Gunasekera S. Ng O. Thomas S. Varcoe R. de Silva C. Barber T. (2020). Tomographic piv analysis of physiological flow conditions in a patient-specific arteriovenous fistula. Exp. Fluids. 61, 253. 10.1007/s00348-020-03085-4

[B17] Gunasekera S. Ng O. Thomas S. Varcoe R. de Silva C. Barber T. (2021). Impact of juxta-anastomotic stent implantation on the haemodynamics within a single representative patient avf. Int. J. Heat. Fluid Flow. 92, 108874. 10.1016/j.ijheatfluidflow.2021.108874

[B18] Huber T. S. Berceli S. A. Scali S. T. Neal D. Anderson E. M. Allon M. (2021). Arteriovenous fistula maturation, functional patency, and intervention rates. Jama. Surg. 156, 1111–1118. 10.1001/jamasurg.2021.4527 34550312 PMC8459303

[B19] Hyde-Linaker G. Barrientos P. H. Stoumpos S. Kingsmore D. B. Kazakidi A. (2022). Patient-specific computational haemodynamics associated with the surgical creation of an arteriovenous fistula. Med. Eng. Phys. 105, 103814. 10.1016/j.medengphy.2022.103814 35781379

[B20] Iori F. Grechy L. Corbett R. W. Gedroyc W. Duncan N. Caro C. G. (2015). The effect of in-plane arterial curvature on blood flow and oxygen transport in arterio-venous fistulae. Phys. Fluids 27, 031903. 10.1063/1.4913754 PMC4368596 25829837

[B21] Jia L. Wang L. Wei F. Yu H. Dong H. Wang B. (2015). Effects of wall shear stress in venous neointimal hyperplasia of arteriovenous fistulae. Nephrology 20, 335–342. 10.1111/nep.12394 25581663

[B22] Jodko D. Obidowski D. Reorowicz P. Jóźwik K. (2017). Blood flows in end-to-end arteriovenous fistulas: unsteady and steady state numerical investigations of three patient-specific cases. Biocybern. Biomed. Eng. 37, 528–539. 10.1016/j.bbe.2017.05.006

[B23] Lok C. E. Huber T. S. Lee T. Shenoy S. Yevzlin A. S. Abreo K. (2020). Kdoqi clinical practice guideline for vascular access: 2019 update. Am. J. Kidney Dis. 75, S1–S164. 10.1053/j.ajkd.2019.12.001 32778223

[B24] Nantakool S. Reanpang T. Prasannarong M. Pongtam S. Rerkasem K. (2022). Upper limb exercise for arteriovenous fistula maturation in people requiring permanent haemodialysis access. Cochrane Database Syst. Rev. 10, CD013327. 10.1002/14651858.cd013327.pub2 36184076 PMC9527110

[B25] Pike D. Shiu Y.-T. Somarathna M. Guo L. Isayeva T. Totenhagen J. (2017). High resolution hemodynamic profiling of murine arteriovenous fistula using magnetic resonance imaging and computational fluid dynamics. Theor. Biol. Med. Modell. 14 (5), 5. 10.1186/s12976-017-0053-x PMC5360029 28320412

[B26] Ramuzat A. How T. Bakran A. (2003). Steal phenomenon in radiocephalic arteriovenous fistula.: *in vitro* haemodynamic and electrical resistance simulation studies. Eur. J. Vasc. Endovasc. Surg. 25, 246–253. 10.1053/ejvs.2002.1842 12623337

[B27] Rangel J. F. Santos W. B. d. A. Costa T. H. d. C. de Bessa K. L. Melo J. D. D. (2023). Pressure analysis in rigid and flexible real arteriovenous fistula with thickness variation *in vitro* . J. Funct. Biomater. 14, 310. 10.3390/jfb14060310 37367274 PMC10299082

[B28] Remuzzi A. Ene-Iordache B. (2013). Novel paradigms for dialysis vascular access: upstream hemodynamics and vascular remodeling in dialysis access stenosis. Clin. J. Am. Soc. Nephrol. 8, 2186–2193. 10.2215/cjn.03450413 23990161 PMC3848396

[B29] Rosado-Toro J. A. Philip R. C. Dunn S. T. Celdran-Bonafonte D. He Y. Berceli S. A. (2022). Functional analysis of arteriovenous fistulae in non-contrast magnetic resonance images. Comput. Meth. Prog. Bio. 222, 106938. 10.1016/j.cmpb.2022.106938 35738094

[B30] Sadaghianloo N. Contenti J. Dardik A. Mazure N. M. (2019). Role of hypoxia and metabolism in the development of neointimal hyperplasia in arteriovenous fistulas. Int. J. Mol. Sci. 20, 5387. 10.3390/ijms20215387 31671790 PMC6862436

[B31] Shahinian V. B. Zhang X. Tilea A. M. He K. Schaubel D. E. Wu W. (2020). Surgeon characteristics and dialysis vascular access outcomes in the United States: a retrospective cohort study. Am. J. Kidney Dis. 75, 158–166. 10.1053/j.ajkd.2019.08.001 31585684

[B32] Shahverdyan R. Beathard G. Mushtaq N. Litchfield T. F. Vartanian S. Konner K. (2021). Comparison of ellipsys percutaneous and proximal forearm gracz-type surgical arteriovenous fistulas. Am. J. Kidney Dis. 78, 520–529.e1. 10.1053/j.ajkd.2021.01.011 33662481

[B33] Shiu Y.-T. Rotmans J. I. Geelhoed W. J. Pike D. B. Lee T. (2019). Arteriovenous conduits for hemodialysis: how to better modulate the pathophysiological vascular response to optimize vascular access durability. Am. J. Physiol. Ren. 316, F794–F806. 10.1152/ajprenal.00440.2018 PMC6580244 30785348

[B34] Sivanesan S. How T. Bakran A. (1998). Characterizing flow distributions in av fistulae for haemodialysis access. Nephrol. Dial. Transpl. 13, 3108–3110. 10.1093/ndt/13.12.3108 9870474

[B35] Suqin L. Mingli Z. Shiteng S. Honglan M. Lan Z. Qihong N. (2020). Assessment of the hemodynamics of autogenous arteriovenous fistulas with 4d phase contrast-based flow quantification mri in dialysis patients. J. Magn. Reson. Imaging 51, 1272–1280. 10.1002/jmri.26936 31584228

[B36] Tordoir J. H. M. Zonnebeld N. van Loon M. M. Gallieni M. Hollenbeck M. (2018). Surgical and endovascular intervention for dialysis access maturation failure during and after arteriovenous fistula surgery: review of the evidence. Eur. J. Vasc. Endovasc. 55, 240–248. 10.1016/j.ejvs.2017.12.001 29307757

[B37] Van Canneyt K. Pourchez T. Eloot S. Guillame C. Bonnet A. Segers P. (2010). Hemodynamic impact of anastomosis size and angle in side-to-end arteriovenous fistulae: a computer analysis. J. Vasc. Access. 11, 52–58. 10.1177/112972981001100111 20119922

[B38] Venkat Ramanan S. Prabhu R. A. Rao I. R. Chawla A. Shenoy S. V. Nagaraju S. P. (2022). Outcomes and predictors of failure of arteriovenous fistulae for hemodialysis. Int. Urol. Nephrol. 54, 185–192. 10.1007/s11255-021-02908-5 34095992 PMC8732889

[B39] Wang Y. Wang Y. Li S. Aziz A. U. R. Liu S. Qin K. (2018). The analysis of wall shear stress modulated by acute exercise in the human common carotid artery with an elastic tube model. Comput. Model. Eng. Sci. 116, 127–147. 10.31614/cmes.2018.03985

[B40] Yang C.-Y. Li M.-C. Lan C.-W. Lee W.-J. Lee C.-J. Wu C.-H. (2020). The anastomotic angle of hemodialysis arteriovenous fistula is associated with flow disturbance at the venous stenosis location on angiography. Front. Bioeng. Biotechnol. 8, 846. 10.3389/fbioe.2020.00846 32793578 PMC7390971

