# Supplementary material for: Adjusting blood redistribution to suppress flow disturbances of hemodialysis arteriovenous fistula: a computational fluid dynamics analysis
Source: Front Bioeng Biotechnol. 2025 Mar 20;13:1551993. doi: 10.3389/fbioe.2025.1551993 (PMC11965668; doi:10.3389/fbioe.2025.1551993)
Supplement: Supplementary file 1 [file DataSheet1.pdf]

Supplementary Material

1 SUPPLEMENTARY FIGURE

Figure S1 shows the results of sensitivity to meshing elements number. The flow velocities corresponding to five levels of mesh densities (I,II,III,IV,V) were calculated and relative errors are compared by calculating the percentage of velocity variations between two consecutive meshing levels. The meshing level III is selected as the relative error ε_2 is less than 5 %.

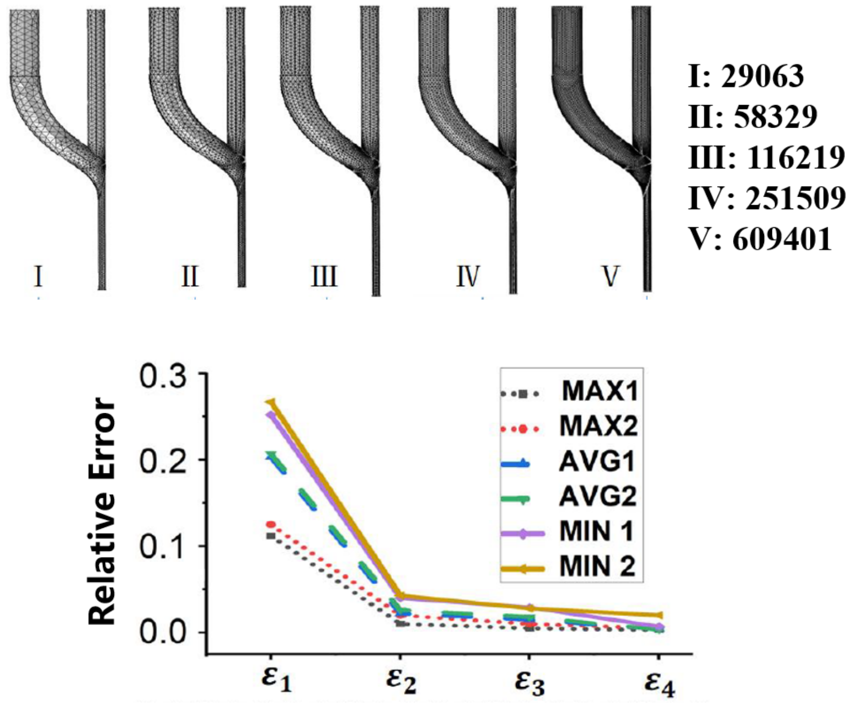

Figure S1. AVF meshing with different elements and the calculation of relative error.
